# Supplementary material for: Are carbon emissions trading and green financial instruments synergistic? -Comprehensive quantitative research based on content analysis
Source: PLoS One. 2024 Mar 7;19(3):e0298601. doi: 10.1371/journal.pone.0298601 (PMC10919601; doi:10.1371/journal.pone.0298601)
Supplement: S1 Data — (ZIP) [file pone.0298601.s001.zip › Data/地级市Z_F工作报告.zip!/╜¡╦╒/─╧═¿╩╨/└┤╫╘╣1⁄2╓┌║┼í╛┬φ┐╦╩2╛▌═°í┐_2005.docx]

各位代表：

　　现在，我代表市人民政府向大会作工作报告，请予审议，并请市政协各位委员和其他列席人员提出意见。

　　一、2004年工作回顾

　　2004年，中央作出落实科学发展观、加强和改善宏观调控的一系列决策部署，我市经济社会发展面临新形势、新机遇、新挑战、新要求。在省委、省政府和市委的正确领导下，按照市委确定的争当全省江北"两个率先"排头兵的奋斗目标和市十二届人大二次会议提出的工作任务，市人民政府积极推进、狠抓落实，全市上下开拓创新、攻坚克难、扎实工作，国民经济在高平台上强劲增长，各项社会事业全面发展，圆满完成年度目标计划，实现了跨越发展的良好开局。

　　（一）国民经济继续强劲增长，产业发展增幅高于全省平均水平。继上年实现"双超"历史性突破后，全市实现生产总值1226亿元，按可比价计算增长15.6%，实现了增幅高于全省平均水平的突破，在长三角16个城市和全省各市中的位次分别由上年的第12位、第6位前移到第7位和第4位。其中一、二、三产业分别增长5.3%、19.5%、14.1%，均高于全省平均水平（以上均为初步核算数），表明我市产业发展融入苏南的进程加快。完成地方一般预算收入52.59亿元，增长26.1%（原口径财政总收入138.72亿元，增长20.8%），财政收入增幅高于生产总值增幅。农业结构调整成效明显，粮食总产增长3.2%，亩产超3000元的中高效农田面积增长7%，畜牧业克服市外高致病性禽流感影响保持平稳增长，海洋捕捞效益创8年来新高，获国家认定的无公害农产品、绿色食品数均居苏中第一，项目农业建设强势推进，"三资"开发农业投资额增长50.1%，创历史新高。预计全部工业实现增加值577.55亿元，增长19.4%；规模以上工业增加值增幅和高新技术产业产值增幅均居全省第二。建筑业完成施工产值580亿元，在全国地级市中名列前茅，增长19.3%。服务业保持健康发展态势。社会消费品零售总额384亿元，增长16%；旅游总收入增长20%，信息服务业发展加快；全市港口货物吞吐量达7218万吨，增长44.1%。县域经济快速发展，六个县（市）生产总值全面超百亿元、五个县（市）财政总收入超十亿元，其中通州市、海门市生产总值超过两百亿元。在经济总量强劲增长的同时，发展质态提高，能源消耗和污染指数下降。二、三产业增加值占全市生产总值比重达87.9%，提高1.7个百分点。高新技术产业产值在规模以上工业产值中占比提高2.7个百分点；规模以上工业企业实现利润增长35%，利税首次超过百亿元；规模以上工业经济效益综合指数达164%，为历史最好水平；大力实施名牌战略，我市产品实现了中国名牌零的突破。万元生产总值综合能耗为1.31吨标准煤，低于全国和全省水平。环境质量稳步提高，我市在全国47个环保重点城市"环境综合整治定量考核"中排名跃居第五，名列"全国污染控制较好城市"第一。

　　（二）投资结构更趋优化，重大项目、江海开发、接轨上海取得新突破。按照国家宏观调控的要求，把握投资方向，继续猛攻带动力强的产业项目和基础性重大项目，预计完成全社会固定资产投资605亿元，增长35%，其中规模以上投资增长39.1%。71个市级重点建设项目完成投资额125.7亿元，占全社会固定资产投资的20.8%；其中32个项目开工建设，24个项目竣工投产。全年完成工业投入400.37亿元，占全社会固定资产投资的66.2%；规模以上工业投入297亿元，增长69.4%。农村水利建设投入加大，政府主导投入增长119%。重大交通基础设施建设加快。苏通大桥建设累计完成投资24.8亿元，占总投资的36.7%，主桥塔墩浇注完成，苏通大桥北接线、盐通高速公路和其它干线公路建设均大幅超额完成计划目标，通启高速公路提前一年建成通车，通常汽渡下迁通渡，新长铁路南通段开通客运，宁启铁路海安段全线贯通。沿江开发、江海联动取得重大进展，30项重点工作全部完成年度目标。在全省率先实现沿江岸线统一管理，建立了涉岸项目准入、退出机制。沿江岸线整合、码头开发取得突破，狼山港三期水工工程、如皋港阳鸿石化5万吨级码头竣工。海港开发建设拉开序幕，洋口港综合开发工程抓紧推进，吕四港大唐电厂前期工作全部就绪。接轨上海取得新突破。沪崇苏大通道建设工程已获国务院批准，我市崇启、崇海大桥建设前期工作完成年度目标，上海至崇明隧桥工程已正式破土动工，为南通接轨上海增添了新的优势；与上海市静安区、杨浦区结为友好区市，在科技、人才、经贸、农业、旅游等方面的接轨取得实质性进展。与苏州市跨江合作向纵深发展。

　　（三）改革攻坚成效显着，争创江苏民营经济第一大市首战告捷。国有经济布局战略性调整和国有企业战略性改组任务基本完成。全年市属企业改制退出公有资本7.06亿元，理顺劳动关系1.86万人。整合市属工贸系统国有资产经营公司，国有资产监督管理体制改革进一步深化。对市区财政体制实行重要改革，对229家市属企业实行属地管理，完善和理顺市、区两级政府管理职能，调动各方加快发展的积极性，增强了整体发展合力。企业上市取得重大进展，3家企业通过中国证监会上市首发审核。继完成生产经营型、行政管理型事业单位改革之后，社会公益型事业单位改革全面启动，事业单位改革进程继续保持省内领先。按照省部署实施市、县政府机构改革，开展乡镇综合改革试点。紧紧围绕争创江苏民营经济第一大市首战必胜的目标，落实扶持民营经济发展的各项政策，大力营造有利于民众创业的社会环境，分类指导推进，强化考核奖惩。民营经济总量在全省的位次前移一位，私营企业数等6项主要指标增幅均居全省首位；板块经济规模进一步壮大，营业收入超10亿元的重点特色板块达12个；民营经济增加值占全市生产总值的比重达41.1%，提高4个百分点。

　　（四）走向世界迈出大步，开放型经济再创佳绩。积极探索开放新路，成功举办南通对外开放史上具有首创性、突破性和战略性意义的首届世界大城市带发展高层论坛，城市对外知名度和影响力大幅提升。继续强化招商攻势，预计全年新批注册外资37.82亿美元，增长63.7%；注册外资实际到帐额11.04亿美元，按原口径折算接近翻番，两项指标完成实绩均列全省第四。利用外资质量提高，结构进一步优化。新批总投资超千万美元的大项目152个，接近苏中三市的一半，协议注册外资达23.7亿美元，占全市总额的62.7%；南通青山电子、高丽钢帘线等投资超亿美元的重量级项目成功落户；新批外资项目平均单体规模达366万美元，比上年增加111万美元；11个省级以上开发区新批注册外资、注册外资实际到帐额和新批总投资超千万美元的大项目分别占全市的66.4%、77%和55%。对外贸易持续攀升，预计全年进出口总额67亿美元，增长30%，其中出口43亿美元，增长31%；外贸依存度达45.2%，提高2.7个百分点。外经合作继续保持全省第一，对外承包劳务营业额6.1亿美元，增长28%。海关、国检、海事、边检等单位实行整体联动，口岸环境不断改善。外事、侨务、对台事务等工作在扩大开放中发挥了积极作用。

　　（五）城市建设快速推进，"五城同创"取得阶段性成效。以中心城市建设为龙头，继续实施城建大推进，全力塑造"中国近代第一城"新形象，城市化进程加快。认真搞好规划编制，全面完成城市总体规划调整和县（市）域城镇体系规划、县（市）城总体规划、省市重点中心镇总体规划及部分试点村村庄集居建设规划编制，编制完成城市绿地系统规划等16项专项规划。新城区建设加速推进，行政中心、南通大学一期工程投入使用，体育会展中心开工建设。主城区濠西路改造等30项重点工程全面完工，污水处理厂扩容等又一批城市基础设施开工建设。认真实施市十二届人大二次会议关于"五城同创"的议案，以优化城市人居环境为着力点，动员和组织社会各方力量，强力推进"八大基础工程"，以创建促管理，重点实施"五横五纵"道路综合整治工程、"3482"整治工程和市容环境整治"梳理行动"，有序推进老旧住宅小区综合改造，加大市区骨干河道、水系整治力度，进一步完善"两级政府、三级管理、四级网络"的管理体制，落实长效管理措施，城市管理水平跃上新台阶。市区创建国家环境保护模范城市通过省级调研和验收，创建国家卫生城市通过省级调研。濠河风景区成为独具南通特色的靓丽风景和城市名片。六县（市）城和中心镇建设步伐加快，基础设施进一步完善，城镇集聚和辐射能力有所增强；继续实施乡镇区划调整，撤并减少8个乡镇。

　　（六）社会事业协调发展，精神文明和民主法制建设得到加强。大力推进科技工作，再获"全国科技进步先进市"称号。列入国家级火炬计划项目数等15项科技单项工作位居全省前列，省级以上科技项目立项数创历史新高，新增1个国家级特色产业基地。一批哲学社会科学成果获奖。高度重视人才队伍建设，人才开发力度进一步加大。教育事业再创佳绩。全面通过省中小学布局调整检查验收，提前一年基本普及高中教育，高考本科录取率等10项指标列全省第一。认真实施市十二届人大二次会议关于加快职业教育发展的议案，组建南通市中等职业教育集团，职业教育、成人教育、终身教育等发展加快。南通大学正式挂牌成立并对外招生，高等教育揭开了历史新的一页。积极推进文化大市建设，群众文化活动活跃，文博馆群建设步伐加快，文物保护工作得到加强，文艺、新闻精品生产再获丰收，广播电视事业快速发展，有线电视实现了村村通。省十六运筹备工作有序推进，成功承办全国击剑锦标赛，南通籍运动员在第28届奥运会和第12届残奥会上喜获2金1银1铜，全民健身体系进一步健全。扎实推进未成年人思想道德建设60件实事，成功承办首届中国公民道德论坛，精神文明建设"南通现象"被中宣部确定为全国公民道德建设重大典型。公共卫生体系建设加快，初步形成了新型农村卫生工作体系和全市疾病预防控制、卫生监督、医疗救治网络及突发公共卫生事件应急防治体系。人口与计划生育工作继续加强，计划生育家庭奖励扶助试点工作进展顺利，计划生育率99%以上，连续7年实现人口负增长。统计、气象、民族、宗教、防震、无线电管理、档案、保密、地方志等工作进一步加强。继续实施依法治市"五年规划"，全民法律素质和机关依法行政水平进一步提高，基层民主法治建设加速推进，超额完成市、县（市）区两级"民主法治示范村（社区）"建设目标。高度重视人民来信来访，解决了一批重大疑难信访问题。深入推进平安南通建设和全省最安全城市创建工作，社会矛盾纠纷大调解机制和社会治安大防控体系进一步健全，全市万人发案率在全省保持较低水平，重大案件破案率、矛盾纠纷调处率在全省领先，社会公众安全感满意率全省第一。强化安全生产和防灾减灾工作，事故起数和死亡人数连续3年"双下降"。深入开展反渗透、反邪教斗争，国家安全工作继续强化。全民国防教育进一步加强，人民防空综合水平在全省领先，征兵、民兵预备役和双拥工作均取得可喜成绩。

　　（七）城乡居民生活质量进一步提高，为民办实事项目完成情况良好。按照构建和谐社会的要求，更加重视解决民生问题。认真落实促进就业再就业的各项政策措施，再就业率达67.4%，年末城镇登记失业率为3%。加大社会保险依法扩面征缴力度，市区实行社会保险统筹。市区居民人均可支配收入突破万元，达10936元，增长14%；预计农民人均纯收入4920元，增长12%，城乡居民收入"九五"以来首次同时实现两位数增长。深化农村税费改革，农民人均减负增收43元。年末城乡居民储蓄存款余额1017.26亿元，增长15.3%。切实解决农村基础设施建设和土地征用、拆迁安置、企事业单位改制中涉及群众切身利益的问题。市区全面实行征地补偿和被征地农民基本生活保障，全市农民集体土地征用补偿款全部发放到位。加强红十字和慈善工作，积极开展"献爱心、送温暖"活动。加大扶贫和老区开发力度。妇女、儿童、老龄、关心下一代工作和残疾人事业取得新成绩。认真履行承诺，12件20项实事完成情况良好。其中提前或超额完成的14项：（1）全年提供城镇就业岗位16.35万个，5.6万名城镇失业人员实现就业再就业，净增就业人数4万人，分别超过计划63.5%、60%、21.2%；（2）农村劳动力转移新增12.1万人，超过计划102%；（3）启海区域供水工程前期工作全部就绪，全市农村改水受益人口新增53.25万人，超过计划56.6%；（4）全市建成278.96万亩无公害农产品生产基地和94个无公害畜产品基地，分别超过计划39.5%、56.7%；（5）12026户、27462间农村草危房改造任务提前半年完成；（6）市区低价位商品房供应70万平方米，超过计划10万平方米，山芝庄棚户区居民迁居安置工作得到落实；（7）投入4000多万元，对市区60多个老旧住宅小区进行了综合改造，对道路、路灯等设施进行了修复，1万户居民自来水一户一表工程提前完成；（8）对8.6万平方米老小区实施了准物业管理；（9）市区人均新增公共绿地1.35平方米，超过计划0.15平方米；（10）在村村通公路的基础上，提高农村公路等级，新建和改造农村公路2268公里，超过计划128公里，新建和改造农村桥梁501座，超过计划140座；加快农村撤渡建桥步伐，在全省第一个实现乡镇无渡口；（11）全市城镇职工基本医疗保险参保面达94.8%，超过计划4.8个百分点，城乡社区卫生服务站普及率达83.5%，超过计划3.5个百分点；（12）城市、农村低保标准均提高了10%，扶持贫困户15000户，脱贫率达61%，发动各方面与5772名重点优抚对象结对帮扶；（13）农村"五保"老人集中供养率达44%，年人均供养标准提高30元，达到1670元，向下肢残疾者赠送轮椅2000辆；（14）110、119、122实行了"三台合一"，整合110社会联动资源、设立社区警务室任务全面完成。按计划如期完成的4项：（1）"肉菜粮放心工程"全面实施，大型农贸市场全面实行了食品质量安全准入制度；（2）市区城闸大桥按期开工建设；（3）市区濠河周边污水截流工程基本完成，对濠河进行了彻底疏浚；（4）市区公交车新增126辆，线路增加14条，公交站台新增450个，70岁以上老人免费乘坐市内公交车已经实施。未能按计划完成的2项：（1）因宏观调控对投资主体的影响，西北片区域供水工程未能完成1亿元的投资计划；（2）通州、启东未能全面启动新型农村合作医疗工作，全市新型农村合作医疗覆盖率为65.4%，与目标还差4.6个百分点。

　　（八）自身建设不断加强，政府效能有了新的提高。切实加强政风建设，政府领导班子成员带头，就转变经济增长方式等重大问题，组织开展了40多个专项和系列调研活动，推动了各项工作的落实。完善政府工作规则和决策程序，主动听取并积极采纳市人大、市政协意见和社会各界建议。大力推行政务公开，决策透明度和社会参与度有了新的提高。狠抓重点工作推进落实，强化了重大项目办公日制度和工业经济、民营经济、利用外资"月公布、季讲评"的推进机制。按照争创全省最佳办事环境的要求，加快政府职能转变，加强机关作风建设，全面贯彻《行政许可法》，市级行政许可事项清理速度全省最快，精简率达78.5%，人民群众和境内外投资者对机关作风的满意度进一步提高。自觉接受市人大及其常委会的法律监督、工作监督和市政协的民主监督，认真执行人大及其常委会决议，切实抓好人大议案和人大代表建议、批评、意见以及政协委员提案的办理工作，市人大代表和政协委员满意基本满意率分别达95.5%、94.8%。认真落实党风廉政建设责任制，强化警示教育，严格执行领导干部述职述廉制度和重大工程预决算、招投标等制度，审计监督进一步加强。

　　各位代表，过去的一年矛盾多、困难多、挑战多、发展平台高，全市能够实现跨越发展的良好开局来之不易。这是在省委、省政府和市委的正确领导下，全市人民团结一致、迎难而上、艰苦奋斗的结果。在此，我谨代表市人民政府向全市广大干部群众，向所有社会主义劳动者和社会主义事业建设者，向市人大代表、市政协委员和离退休老同志，向人民解放军、武警驻通部队官兵和公安干警，向各民主党派、工商联、各人民团体和各界人士，向大力支持南通现代化建设的部、省驻通单位，向所有关心支持南通发展的港澳台同胞、海外侨胞和国际友人，表示崇高的敬意和衷心的感谢！

　　各位代表，在成绩面前我们也清醒地看到，我市经济社会发展中还面临不少矛盾和问题，政府工作还存在着许多薄弱环节。经济增长方式由粗放型向集约型转变的任务还很艰巨；财政收入的增幅还不够高；随着城市化进程加快，城乡基础设施建设相对滞后，中心城市对县（市）城、中心镇的带动和辐射作用还不强，产业发展的承载能力有待提高；社会事业发展的活力还不够足，农村教育、文化、卫生等社会事业发展仍不够平衡；社会保障程度还不高，部分群众收入增长还不快，农民增收的基础比较脆弱。对照发展新要求，政府职能转变和驾驭市场经济的本领在某些方面还不适应；一些部门的服务意识和创新争先意识还不强，真抓实干的作风还不硬，一些公务员依法行政的水平还不高，机关行政效能和服务水平有待进一步提高。对此，我们将高度重视，采取扎实措施，认真加以解决。

　　二、2005年主要任务

　　今年是全面实现"十五"计划目标、衔接"十一五"发展的重要一年，也是朝着"两个率先"目标迈进的关键一年。政府工作要以邓小平理论和"三个代表"重要思想为指导，认真贯彻党的十六大、十六届三中、四中全会和中央、省、市经济工作会议精神，全面落实科学发展观，紧紧围绕争当全省江北"两个率先"排头兵的奋斗目标，按照市委确定的"依托江海，崛起苏中，融入苏南，接轨上海，走向世界，全面小康"的总体思路以及相关工作部署，深化改革，扩大开放，着力转变经济增长方式，继续猛攻有效投入，大力推进结构调整，加快市场化、工业化、城市化、国际化进程，不断提高城乡人民生活水平，实现好、维护好、发展好人民群众的根本利益，推动南通既快又好地发展，实现更高起点上的新跨越。

　　今年全市经济社会发展的主要预期目标是：全市生产总值增长13%以上；地方一般预算收入增长15%以上；全社会固定资产投资增长25%以上；社会消费品零售总额增长13.5%以上；注册外资实际到帐额增长25%以上；城镇居民人均可支配收入增长10%以上，农民人均纯收入增长8%以上；城镇登记失业率控制在4%以内；人口自然增长率控制在零以下。

　　根据以上总体要求和发展目标，重点抓好以下工作：

　　（一）突出产业结构优化升级，继续猛攻有效投入。我市正处于工业化中期、城市化加速期、经济国际化提升期，面临扩张总量、提升质态的双重任务。为此，我们必须立足发挥比较优势，继续猛攻有效投入，加快转变经济增长方式，注重用增量带动调整，在调整中加速发展，继续深入实施以工兴市第一方略，走新型工业化道路，加速服务业发展，增强服务业对产业发展的支撑力，进一步提高农业综合生产能力，提高二、三产业比重，尽快融入苏南板块。全社会固定资产投资力争达到756亿元，其中规模以上投资占比超过75%。二、三产业增加值占全市生产总值比重提高到89%以上，其中工业增加值占全市生产总值比重提高到48%以上；服务业增加值增长13%以上，服务业贡献率要有新的提高。发挥江海优势，优化产业布局。充分用好"靠江、靠海、靠上海"的独特优势，深入实施《南通市沿江开发详细规划》，大力发展临港产业，全力打造现代纺织服装、船舶修造、电力能源、电子信息、冶金、精细化工和石油化工、新材料和造纸、食品医药等支柱产业，促进沿江沿海两大产业带建设。加快海港基础设施建设，推进洋口港综合开发和吕四港开发工程。强化规划引导，加快建立招商、开发利益共享机制，促进企业、项目向省级以上开发区和重点工业园区集聚，推动开发区和园区向集约化、特色化、专业化方向发展，构建"集约开发、集中布局、集聚发展"的产业新格局。引导各地因地制宜，壮大优势产业、优势集群，做粗拉长产业链，形成具有特色竞争力的产业板块。推进科技创新，提升产业层次。围绕提高产业竞争力，以科技创新推动产业向"高附加值、高科技含量、低消耗、低污染"转型。进一步完善激励政策，大力引进高新技术，推动科技水平的提高。深入实施科技企业孵化器建设工程、发展高新技术产业"456"工程，确保高新技术产业产值增长30%以上。引导和支持工业企业加大技改投入。继续实施名牌带动战略和差别化扶持政策，加快培育一批年销售超30亿元、具有国际竞争力的大企业、大集团。积极扶持"专、精、新、特"成长型企业发展，增强产业配套能力。以提高装备水平、拓展市场空间为重点提升建筑业，着力在培育大企业、承揽大项目上取得新突破，力争全市新增一级以上资质企业5-6家，其中特级资质企业1家，施工产值突破650亿元。加快发展服务业，提高产业发展支撑力。把发展服务业放在更加突出的位置，优先发展现代金融、现代物流、信息服务等新兴服务业，积极发展传统服务业特别是劳动密集型服务业。加快提升商贸业，调整优化商贸业布局。加快以板块经济为特色的大市场建设，进一步做大、做强9个成交额超10亿元的专业市场。加快发展以港口、交通枢纽为依托的现代物流业，规划建设港口综合物流园区。完善旅游业发展规划，加快发展以江海风光为特色的旅游业，积极融入"苏浙沪"旅游圈，力争全年旅游总收入达64亿元。积极推进社会公共领域信息化。加强政策引导，改善消费环境，培育消费热点，增强消费对经济增长的拉动作用。精心组织重大、重点项目实施，推动结构升级落到实处。把符合国家产业政策、牵动发展全局的重大项目紧紧抓在手上，全力推进平均单体投资规模达7.83亿元的93个重点建设项目，特别是王子制纸等超大型、基地型龙头项目。注重抓好重大产业项目储备，加强34个重大产业储备项目的前期工作，力争有更多的大项目进入国家、省重点项目盘子。集约节约利用资源，增强可持续发展能力。正确处理生产、生活、生态的关系，禁止新上高物耗、高能耗、高污染、低产出项目。严格执行国家土地政策，开展新一轮土地利用总体规划修编工作，坚持有序、高效用地，确保用地向重大产业项目和重大基础设施项目倾斜，向国家级、省级开发区倾斜，向老百姓迫切需要解决的实事项目倾斜。建立健全各类项目用地准入、退出机制，提高单位土地投资强度，加大土地利用监管力度，不断提高土地利用率、建筑容积率和投入产出率。继续抓好江海岸线资源整合、整治、开发，严格执行岸线投资强度标准，落实涉岸项目准入、退出制度。大力发展循环经济，加快建设生态市，制定并实施《南通市循环经济规划》和《清洁生产五年行动计划》，积极推进资源综合利用，推行污染全面达标排放和减量排放。

　　（二）突出抓好农民增收，继续加强"三农"工作。坚持把"三农"工作作为全局工作的重中之重，按照统筹城乡发展的要求，发挥工业对农业的支持、反哺作用和城市对农村的辐射、带动作用。继续实施"农民收入倍增工程"，加速农村现代化和全面小康建设。加快农业产业化进程。在高度重视粮食安全的同时，以市场为导向，大力发展项目农业、特色农业、高效农业、品牌农业。加快建设优势特色农产品产业带，建立10个以上市级现代农业示范园区，重点培育60家市级以上龙头企业，不断提高龙头企业与农户的利益关联度。全面推进农业标准化工作和农产品质量体系建设，提高农业机械化水平，建立健全农业社会化服务体系，进一步提高农业土地产出率和农业综合效益，力争今年亩产超3000元的中高效农田占比提高5个百分点。充分利用海洋资源，继续推进"海上南通"建设。加快转移农村劳动力。构建覆盖城乡的农村劳动力供需网络，畅通农村劳动力转移通道。突出抓好劳务输出，大力发展劳务经济，加快区域间、城乡间的内部转移和就地转化，确保农村劳动力转移率提高2个百分点以上，出国劳务总量继续保持全省领先。加快创新农村发展机制。加强对农业的政策支持，提高财政支农比重；加大信贷支农力度，积极促进"三资"开发农业，构建多元化的农村投入机制；全面取消农业税。继续大力发展农民专业合作经济组织和农民经纪人队伍。积极推动城市流通网络向农村延伸。依法搞好征地补偿安置。按照依法、自愿、有偿的原则，积极、稳妥、有序地推进农村集体土地使用权流转，探索让农民从土地增值中长久得益的途径。进一步加大农民负担监管力度，切实维护农民的合法权益。加快农村城镇化进程。按照城乡一体的要求，高标准、高起点搞好新一轮村镇规划，并与新一轮土地利用总体规划相衔接，合理布局农村产业、城镇、村庄和网络设施，着力推进农民建房布局的科学化，提高农民生活质量。加强交通、水利、管网等农村基础设施建设，统筹发展农村社会事业。加快推进绿色南通建设，努力提高林木覆盖率。深入实施农村"清洁村庄、清洁河道、清洁家园"工程，努力优化农村生态环境。

　　（三）保持民营经济蓬勃发展态势，加快经济市场化进程。以争创江苏民营经济第一大市和优化所有制结构为重点，进一步完善社会主义市场经济体制，再创南通体制机制新优势。推动全民创业持续升温。按照"思想彻底解放、政策彻底放宽、服务彻底到位"的要求，全面落实扶持民营经济发展的各项政策措施。鼓励民营企业生产经营国家法律法规没有明确限制的一切产品，支持民间资本进入所有已向外资开放的行业，加快民间资本进入基础设施、公用事业领域。坚持舆论引导、服务推动、考核激励等多策并举，再掀全民创业高潮，确保全市新增个体工商户4万户、私营企业1万家、注册资金150亿元，实现民营经济"一个领先、一个融入、一个提高"，即各项主要指标增幅继续保持省内领先，部分质量指标融入苏南，确保民营经济增加值占全市生产总值的比重提高2个百分点。加快提升民营经济发展层次。在扩张总量的同时，引导民营企业做大做强，提高产业层次和素质。加大对8家销售超10亿元民营企业和科技型、外向型民营企业的扶持力度，着力培育骨干民营企业集群。大力实施"百强民企上市工程"，力争10家企业进入上市辅导期和境外上市轨道，2-3家企业通过证监会审核。依托市场，突出整合，重点打造家纺用品、电动工具、工艺编织、钢丝绳等民营经济板块，提高块状经济专业化、社会化和规模化水平。加强对民营经济的引导和服务。认真落实省、市促进民营经济发展的各项举措，努力为民营经济发展提供优质高效服务。开展民营企业家培训活动，培养和造就优秀民营企业家队伍。加强中小企业信用担保体系建设，深入推进银企合作，积极化解民企融资难题。引导和激励骨干民营企业攀高争先、做大做强，增强核心竞争力，成为全省乃至全国行业的"单打冠军"。深入推进以市场为取向的各项改革。围绕解决影响发展全局的深层次矛盾和问题，增强经济发展的内生动力和体制机制活力，着力形成全面、协调、可持续发展的制度保障。完善国有资产监督管理体制，更好地发挥国有资产效益。落实投融资体制改革措施。加快推进并完成社会公益型事业单位的分类改革，增强社会事业发展活力。加强要素市场和行业协会、商会建设，积极引进境内外会计师、律师事务所等中介机构，提升市场中介服务水平。继续整顿和规范市场经济秩序，加强市场物价、产品质量监管，完善社会信用体系，保障经济健康发展。

　　（四）保持开放型经济跃升态势，加快经济国际化进程。把加快融入经济全球化、区域一体化进程，向国际资本要生产力，作为转变经济增长方式、推动南通跨越发展的重大举措，加速重返江苏开放型经济第一方阵。推动开放型经济实现新跨越。紧紧抓住南通招商引资的战略机遇，继续加大对外宣传推介力度，组织好招商引资重大活动，保持招商引资的强烈氛围和利用外资的强劲态势。立足于提高招商引资成效和利用外资质量，调整完善利用外资工作思路。更加注重把意向外资转化为合同外资，转化为到帐外资，转化为项目产出，力争外资到帐率、项目落地率、开工投产率明显提高。加大对侨资、台资的招商力度，鼓励现有外资企业增资扩股。拓展招商引资领域，努力在利用外资发展特色农业、服务业和推进基础设施建设上取得突破。积极引进国际先进技术，以引智带动引资，以引资促进引智。转变外贸增长方式，增强按国际通行规则发展和保护自己的能力，积极规避国际市场风险，不断优化出口产品结构，大力拓展国际市场，重点提高机电、高新技术产品出口比重，力争千万美元以上出口大户超过80家。完善口岸"大通关"功能，进一步优化出口环境。继续保持外经工作在全省的领先位置。鼓励和引导更多有条件的企业"走出去"，开发利用境外资源。扩大劳务输出规模，努力提高智力型、技术型和管理型劳务输出比重。增创开发区集约发展新优势。完善开发区发展规划，强化产业承载功能，充分发挥对外开放主阵地作用，加快争先晋位进程，推动做大总量、做强规模，把多年形成的基础加速转化为产出能力，提高开发区在全市经济增长中的贡献份额。加速融入长三角一体化新进程。以承办长三角城市经济合作第六次会议为契机，更紧密地参与以上海为龙头的长三角经济合作，深入实施《南通市接轨上海工作纲要》，加强在交通衔接、产业整合、要素共享、旅游一体化等方面的互动，力争在承接制造业转移、加强现代服务业合作等方面取得较大突破。继续推进与苏州市的跨江联动。

　　（五）保持"五城同创"强力推进态势，加快城市化和城市现代化进程。顺应以上海为龙头的长三角大城市群迅速崛起的趋势，高起点统筹推进中心城市与县（市）城建设，优化城市布局体系，继续实施城建大推进，力争城市化率提高2个百分点以上。科学统筹城乡规划，促进区域协调发展。按照科学统筹、城乡一体的要求，启动第四轮城市总体规划的修编工作，超前谋划新一轮跨越发展的空间布局。全面推进控规编制工作，强化规划对城市建设的龙头作用。加快交通基础设施建设，继续拉开大城市发展框架。加大投入，加强市域重大交通基础设施的规划建设和城市道路网络建设。抓紧实施"3511"路桥工程，打通市区路网15条断头路，优化城市路网结构；打通城市对外快速通道，实现城市道路与高速路网的互连互通。继续做好苏通大桥建设的服务保障工作和崇启大桥、崇海大桥开工前期工作，推进苏通大桥北接线建设。做好沿海高速公路建设的前期工作，加快盐通高速公路南通段和沿江公路建设。力争开工建设南通至南通东、南通东至狼山港铁路，完成宁启铁路南通东至启东段建设准备工作。完善中心城市功能，提高承载辐射能力。新城区建设要有新突破。强化国家级经济技术开发区作为新城区重要组成部分的城市功能。加紧推进体育会展中心、南通大学二期工程、新城区商务中心建设，加快形成新城区雏形。继续改造提升老城区。启动历史街区的保护性开发和通吕运河沿线整治的规划工作，推进港闸区和老城区西部的改造。整体规划、高品位推进"五山"风景区综合开发。继续优化濠河周边环境，打造精品，增加亮点。继续加大城市基础设施投入力度，加快推进"西气东输"配套工程，启动狼山水厂、洪港水厂扩容工程。优先发展城市公共交通，加快停车场建设。加快县（市）城和中心镇开发建设，增强要素吸纳、社会服务和经济辐射功能。深入开展创建工作，全面改善人居环境。继续大力推进以创建文明城市为龙头的"五城同创"，争取创建国家卫生城市通过国家级考核验收，创建国家环境保护模范城市通过国家级调研、考核，创建国家园林城市通过省级考核验收，加快申报国家、省历史文化名城。完善城市建设管理体制，强化行政综合执法，进一步提高城市管理水平。继续实施"3410"环境综合整治和"五横五纵"道路综合整治工程，继续解决好影响市容环境的突出问题。拓宽城建筹资渠道，提高经营城市水平。集约利用城市土地资源。合理安排土地开发时序，加大储备地块拆迁力度，适当放大土地出让总量，提高出让效益。通过加大政府投入带动更多的社会投入，拓宽城建筹资渠道。进一步开放和规范房地产市场，鼓励成片改造、规模开发，市区商品房竣工面积不少于150万平方米。

　　（六）统筹发展社会事业，促进社会全面进步。全面实施科教兴市、人才强市战略，加快发展社会事业，加强社会主义精神文明和民主法制建设，实现社会全面进步和人的全面发展。不断提高科教发展水平。支持南通大学加快建设，提升南通高等教育整体实力，充分发挥南通大学服务地方经济和社会发展的重要作用。完善义务教育"以县（区）为主"管理体制，进一步改善办学条件，确保农村中小学完成"六有"工程，并通过省级验收。继续办好学前教育和特殊教育。推进教育现代化工程，力争有1个县（市）区进入省教育现代化县（市）区行列。大力发展职业技术教育，加强校企合作，鼓励多元投资，建立技能型紧缺人才培养培训机制。构建开放式、社会化教育体系，发展成人教育和民办教育，推进办学多元化。标本兼治，治理教育乱收费。构建以企业为主体的科技创新体系，提高企业自主创新能力。建设一批面向企业的公共技术服务平台。加快发展多种形式的科技中介服务机构。强化人才对跨越发展的支撑。继续营造尊重知识、尊重人才的浓厚社会氛围。优化人才结构，大力引进我市急需的产业人才，提高产业人才在全部人才中的占比。充分发挥人才市场和海外留学人员创业园的作用。加快推进文化大市建设。科学推进文化事业单位转型和文化产业升级。大力实施文化精品战略，精心组织文化产品生产，加强基层群众文化活动阵地建设。加快发展文博事业，完成博物苑新馆建设，办好南通博物苑百年苑庆暨中国博物馆事业百年庆典活动，加大文化推介力度，加强文化遗产保护。狠抓广播电视精品生产，提高有线电视覆盖率，力争新增有线电视用户10万户。进一步加强档案和地方志工作。加快完善全民健身体系，抓紧做好全运会和省十六运参赛、办赛工作。切实提高公共卫生及人口与计划生育服务水平。全面构建新型公共卫生体系，加强卫生监督、疾病预防控制、医疗救治体系建设，建立突发公共卫生事件处置机制。加强乡镇卫生所和社区卫生服务体系建设，提高城乡医疗卫生服务水平。深化计生优质服务，稳定低生育水平，提高出生人口素质。加强社会主义精神文明建设。深入实施《公民道德建设实施纲要》，加强和改进未成年人思想道德建设，进一步发扬光大精神文明建设"南通现象"。深入开展群众性精神文明建设活动，积极推进文明城市创建工作，争创全省江北第一文明城市群。继续加强双拥和优抚工作，支持驻通部队建设，再创全国"双拥模范城"。切实加强保密、气象、防震减灾、无线电管理等工作，做好民族、宗教工作。

　　（七）维护人民群众利益，努力构建和谐社会。坚持以人为本，注意协调好各方面利益关系，采取切实有效措施维护社会公平，调动一切积极因素，努力形成全体人民各尽其能、各得其所，全面发展而又和谐相处的社会局面。积极扩大城乡就业。坚持在发展中解决就业问题，认真落实促进就业的各项扶持政策，加快建立城乡一体的劳动力市场，切实加强再就业援助工作，全年提供城镇就业岗位11万个以上。积极发展就业容量大的劳动密集型产业和社区服务业，加大社区公益性岗位开发力度，不断拓宽就业渠道。强化劳动力就业技能培训、信息服务、组织协调，提高劳动者复合技能和创业创新能力。积极探索与户籍制度相适应的就业和失业管理制度，进一步健全就业服务体系。规范劳动用工管理，完善企业职工工资合理增长机制，保证农民工工资及时足额发放，维护广大职工、农民工的合法权益。进一步完善社会保障体系。严格依法推进社会保险全覆盖，及时把进城就业农民纳入各项社会保险范围，切实加强基金征缴，坚持应收尽收、应保尽保，努力构建社会保障安全网。建立社会弱势群体医疗救助机制，完善多层次的医疗保险体系。关心困难群众生产生活，继续办好惠及群众的实事工程。切实解决下岗职工、失地农民、拆迁居民生产生活困难，积极帮助困难家庭解决好看病难、子女上学难问题。把政府救济和社会救助结合起来，积极发展社会福利、社会救济、社会慈善和残疾人事业，鼓励、动员社会各界广泛开展"献爱心、送温暖"活动。做好扶贫和老区开发工作。在广泛征求市民意见的基础上，按照与群众利益密切相关、受惠面广、迫切需要解决、财力可以承受、年内基本可以落实，以及保持延续性的原则，集中有限财力，重点抓好以下10件实事项目：（1）全年实现3.5万名城镇失业人员就业。（2）全市新型农村合作医疗覆盖率达80%；大力推进农村社区卫生服务站建设，覆盖率达85%。（3）加快推进西北片区域供水工程、启海区域供水工程；完成农村改水攻坚任务，农村自来水普及率达95%；完成市污水处理厂二期、江海港区污水处理厂工程，启动观音山污水处理厂一期工程，确保市区污水处理率达到70%。（4）加快市区农贸市场改造升级，改造市区端平桥、新桥等4个农贸市场。（5）市区新建低价位商品房60万平方米。（6）改造农村危桥360座，新建和改造农村等级公路1300公里。（7）全市疏浚25条（段）县级河道和150条（段）乡级河道，切实改善河道水质。（8）城市、农村低保标准提高10%左右。（9）安排专项资金用于送温暖工程，对市属改制企业中有特殊困难的职工发放生活救济金；通过多种渠道，适当提高企业离退休市劳模待遇。（10）全面推行农村部分计划生育家庭奖励扶助制度，对符合条件的农村居民按每人每月50元的标准予以奖励。大力推进法治南通建设。抓紧实施依法治市"五年规划"，制订《法治南通建设实施纲要》年度工作计划，深入开展法制宣传教育，提高全民法治观念和法律素质，积极营造全社会尊法守法、依法维权的良好环境。自觉接受市人大及其常委会的法律监督和工作监督。加强与人民政协、各民主党派、工商联和各人民团体的联系与沟通，积极支持政协履行政治协商、民主监督、参政议政的职能。继续开展"民主法治示范村（社区）"创建活动，深入推进基层民主法治建设，保证人民依法实行民主选举、民主决策、民主管理和民主监督。继续重视老龄事业，切实保障妇女、儿童合法权益，加强青少年保护。认真落实维护社会稳定工作责任制，进一步加强信访工作，提高新形势下化解人民内部矛盾的能力。大力推进平安南通建设，着力巩固社会矛盾纠纷大调解机制和社会治安大防控体系，继续深入开展反渗透、反邪教斗争，依法打击各种犯罪，争创全省最安全城市。建立健全社会预警体系，提高保障公共安全和处置突发事件的能力。高度负责地抓好安全生产和食品卫生安全。

　　三、加强政府自身建设

　　按照党的十六届四中全会精神，切实加强政府自身建设和公务员队伍建设，把"为民、务实、清廉"的要求落实好，努力做到让人民满意。

　　（一）坚持执政为民，建设服务型政府。进一步营造发展经济、善待百姓的氛围，始终坚持把握南通实现"两个率先"、全面建设小康社会的目标要求，以发展为第一要务，高点定位，自加压力，负重奋进，争创一流，使最广大人民群众更快富裕起来。继续争创全省最佳办事环境。加强机关效能建设，强化效能监察。大力优化投资环境和办事环境，切实加强行政审批服务中心、行政效能监察和投诉中心建设，依法保护投资者利益。多做群众急需的事，多做群众受益的事，多做打基础的事，多做对群众利益起长远作用的事。强化公务员的自身学习，切实加强公务员队伍的思想建设、作风建设、职业道德建设，努力提高服务发展、服务基层、服务群众的本领。

　　（二）坚持求真务实，建设责任政府。按照"经济调节、市场监管、社会管理、公共服务"的定位，把落实科学发展观贯穿于政府工作全过程，不断转变政府职能、改进管理方式，突出抓好重大生产力布局、资源集约节约利用、环境保护、维护社会公平、保障公共安全。坚持讲实话、鼓实劲、重实绩、求实效，以好的政风强化狠抓落实第一基本功，真正做到对历史负责，对发展负责，对人民负责。搞好经济普查，加强对全局性、战略性、前瞻性重大问题的研究，做好"十一五"规划编制工作。完善公众参与、专家论证和政府决策相结合的决策机制，加快建立和完善重大问题集体决策制度、专家咨询制度、社会公示和听证制度、决策责任制度。同时，建立健全决策跟踪反馈和评估机制，完善责任与权力相统一的执行机制，严格实行目标管理责任制，严格考核奖惩，强化行政过错责任追究，做到有权必有责、用权受监督、侵权要赔偿、违法须追究。

　　（三）坚持依法行政，建设法治政府。认真贯彻《全面推进依法行政实施纲要》，坚持将依法行政贯穿于政府工作全过程，使政府各项行政管理规范化、法治化。加强电子政务建设，积极推进政务公开，保障公众对公共事务的知情权，提高行政管理透明度。加强和改善行政执法，依法规范和行使行政权力，完善执法监督体制。严格执行行政复议法，完善行政复议工作制度。认真贯彻人大及其常委会的决议、决定，及时主动报告工作，自觉接受人大常委会对政府工作的评议和对政府组成人员的述职评议，高度重视办好人大议案和代表建议、批评、意见以及政协委员提案，让代表、委员满意。

　　（四）坚持从严治政，建设廉洁政府。认真落实党风廉政建设责任制，健全教育、制度、监督并重的惩防体系，严格执行廉洁自律各项规定。大力推进制度创新，使公共权力在社会公开监督和制度制约下运行。严格执行土地批租、工程招投标、政府采购的各项规定，坚决防止暗箱操作，堵塞公权私用的漏洞。进一步规范产权交易行为，防止国有资产流失。严肃财经纪律，进一步加强财政资金预算管理和审计监督。深入推进纠风专项治理工作，切实纠正行业不正之风。对违反廉政规定、侵害公民权益的人和事，坚决查处，决不姑息。认真贯彻落实两个《条例》，坚持法律监督、纪律监督、民主监督、群众监督和舆论监督并举，不断强化权力监督制约机制。

　　各位代表！在更高起点上全力推动南通既快又好发展，是时代的召唤、富民的要求、历史的重任。让我们高举邓小平理论和"三个代表"重要思想伟大旗帜，全面贯彻党的十六届四中全会精神，在省委、省政府和市委的领导下，紧紧依靠和团结全市人民，励精图治，奋发有为，把南通跨越发展大业继续推向前进！
